# Bioaccumulation of titanium dioxide nanoparticles in green (*Ulva* sp.) and red (*Palmaria palmata*) seaweed

**DOI:** 10.1007/s00604-023-05849-1

**Published:** 2023-07-07

**Authors:** Juan José López-Mayán, Blanca Álvarez-Fernández, Elena Peña-Vázquez, María Carmen Barciela-Alonso, Antonio Moreda-Piñeiro, Julie Maguire, Mick Mackey, Monica Quarato, Ivone Pinheiro, Begoña Espiña, Laura Rodríguez-Lorenzo, Pilar Bermejo-Barrera

**Affiliations:** 1 Trace Element, Spectroscopy and Speciation Group (GETEE), Institute of Materials (iMATUS), Faculty of Chemistry, University of Santiago de Compostela, 15782 Santiago de Compostela, Spain; 2 Indigo Rock Marine Research, Gearhies, Bantry, Co. Cork, P75 AX07 Ireland; 3 International Iberian Nanotechnology Laboratory, Av. Mestre José Veiga, s/n, 4715-330 Braga, Portugal

**Keywords:** Bioaccumulation, Titanium dioxide nanoparticles, Seaweed, Alkaline extraction, Single particle inductively coupled plasma mass spectrometry, Electron microscopy with energy dispersive X-ray analysis

## Abstract

A bioaccumulation study in red (*Palmaria palmata*) and green (*Ulva* sp.) seaweed has been carried out after exposure to different concentrations of citrate-coated titanium dioxide nanoparticles (5 and 25 nm) for 28 days. The concentration of total titanium and the number and size of accumulated nanoparticles in the seaweeds has been determined throughout the study by inductively coupled plasma mass spectrometry (ICP-MS) and single particle-ICP-MS (SP-ICP-MS), respectively. Ammonia was used as a reaction gas to minimize the effect of the interferences in the ^48^Ti determination by ICP-MS. Titanium concentrations measured in *Ulva* sp. were higher than those found in *Palmaria palmata* for the same exposure conditions. The maximum concentration of titanium (61.96 ± 15.49 μg g^−1^) was found in *Ulva* sp. after 28 days of exposure to 1.0 mg L^−1^ of 5 nm TiO_2_NPs. The concentration and sizes of TiO_2_NPs determined by SP-ICP-MS in alkaline seaweed extracts were similar for both seaweeds exposed to 5 and 25 nm TiO_2_NPs, which indicates that probably the element is accumulated in *Ulva* sp. mainly as ionic titanium or nanoparticles smaller than the limit of detection in size (27 nm). The implementation of TiO_2_NPs in *Ulva* sp. was confirmed by electron microscopy (TEM/STEM) in combination with energy dispersive X-Ray analysis (EDX).

**Graphical abstract:**

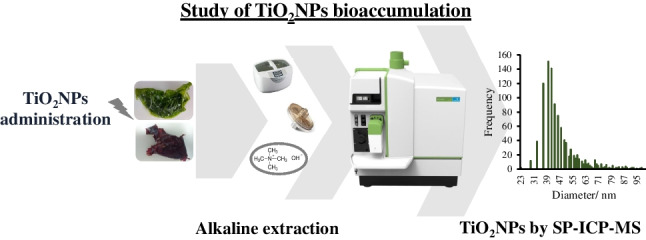

**Supplementary Information:**

The online version contains supplementary material available at 10.1007/s00604-023-05849-1.

## Introduction

Titanium dioxide (TiO_2_), known as titanium white or titania, is a metallic oxide widely used in the industry as a pigment due to its high refractive index, brightness, and resistance to discoloration [1, 2]. Despite the widespread use of TiO_2_, the European Food Safety Authority (EFSA) concluded that it cannot be considered safe for human health [3], and the European Union has implemented a regulation to ban the use of food additive E-171 since February 2022 [4]. TiO_2_ had been also classified as possibly carcinogenic to humans by the International Agency for Research on Cancer (IARC) [5].

There is consensus in scientific studies that indicate that the rise in the use and production of nanomaterial (NMs) increases their release into the environment (through air, soil, and water) [1]. Then, titanium dioxide nanoparticles (TiO_2_NPs) can enter the marine systems and interact [6] and endanger the biota [7]. There are a couple of published review papers with information about the nanotoxicity of TiO_2_NPs in marine organisms [8, 9], and several recent studies have evaluated the interaction and toxic effects of TiO_2_NPs in marine microalgae *Dunaliella tertiolecta* [10], *Dunaliella salina*, and *Chlorella* sp. [11]. Bhuvaneshwari et al. [12] also studied the trophic transfer of 25 nm TiO_2_NPs from *Dunaliella salina* to crustacean *Artemia salina* in terms of toxicity and accumulation. However, information is still lacking about the accumulation and effects of NPs in marine macroalgae (seaweed).

Seaweed are bioconcentrator organisms, and their use is increasing in the food industry not only as gelling agents or food supplements but also as a potential source of fiber, nutrients, and trace elements [13, 14]. Therefore, they can act as a vector of NPs through the food chain and can be transferred to humans. Some authors studied the presence and the bioaccumulation of heavy metals in seaweed [15]. Ryan et al. [16] studied the distribution of Pb, Zn, As, Cd, Co, Cr, Cu, Mn, and Ni in *Polysiphonia lanosa*, *Ascophyllum nodosum*, *Fucus vesiculosus*, and *Ulva* sp. from the south of Ireland. Jarvis et al. [17] reported the accumulation of Cu, Ni, Pb, Cd, and Zn in *Ulva lactuca* and *Agardhiella subulate* exposed to the metals individually and in mixtures (100 and 1000 μg L^−1^) for 48 h. The distribution of metals in both seaweed species changed with increasing metal exposure concentrations. *U. lactuca* exposed to single metals showed higher concentrations as compared to exposure to metal mixtures, with Cu and Zn generally accumulating to the highest magnitudes. Carvalho et al. [18] studied the presence of heavy metals including Ti in different parts (base, stipe, reproductive organs, and growing tips) of *Fucus vesiculosis* by energy-dispersive X-ray fluorescence. Misher et al. evaluated the metal uptake (including Ti) of *Caulerpa racemosa* [19], *Ulva lactuca* [20], *Gelidium abbottiorum* [21], and *Plocamium corallorhirza* [22] from the coast of South Africa. Desideri et al. [23] measured 21 elements (including Ti) in 14 edible seaweed bought in Italy, and Corrias et al. [24] reported the environmental evaluation of heavy metals and metalloids (Al, As, B, Ba, Be, Cd, Co, Cr, Cu, Fe, Hg, Mn, Mo, Ni, Pb, Sb, Se, Sn, Sr, Te, Ti, V, and Zn) in seaweeds (*Padina pavonica* and *Cystoseria mediterranea*) from the Mediterranean sea, where Ti was found in all seaweed specimen from all sampling zones (*n* = 3). There are also a few studies about the interactions and uptake of AgNPs by *Ulva lactuca* [25] and CuONPs by *Ulva lactuca* and *Agardhiella subulate* [26], and *Ulva rigida* [27] after short term exposure (less than 72 h).

However, to the best of our knowledge, there are no reports available in the scientific literature about bioaccumulation assays in edible macroalgae exposed to TiO_2_NPs. This research work aims to study the bioaccumulation of TiO_2_NPs in two red and green edible seaweeds (*Palmaria palmata* and *Ulva* sp.) exposed to different sizes (25 and 5 nm in diameter) and concentrations of citrate coated-TiO_2_NPs for 28 days. The variation in the trend of total titanium and nanoparticulate content (number and TiO_2_NPs size) with exposure time was evaluated by inductively coupled plasma-mass spectrometry (ICP-MS) and single-particle-ICP-MS (SP-ICP-MS), respectively. TEM/SEM combined with EDS was used to find the localization of TiO_2_NPs in some of the specimens studied.

## Materials and methods

### Instrumentation

For total titanium quantification, an inductively coupled plasma-mass spectrometer (ICP-MS) NexION^®^ 2000 (PerkinElmer, Waltham, MA, USA) equipped with dynamic reaction cell (DRC) technology was used. The Syngistix™ Nano Application 2.5 software (PerkinElmer) allows working in the single-particle mode (SP-ICP-MS) to obtain TiO_2_NPs concentration and size distributions. An Ethos Plus microwave lab station (Milestone, Bergamo, Italy) was used for sample acid digestion. An USC 300 TH ultrasound water bath (ultrasound frequency of 45 kHz, 80 W) from VWR (Radnor, PA, USA) was used for TiO_2_NPs isolation.

The analysis of the size and shape of the TiO_2_NPs stocks was performed using JEOL 2100 Transmission Electron Microscopy (TEM) operating at 200 kV (Izasa Scientific, Carnaxide, Portugal) for 25 nm TiO_2_NPs and FEI Titan Cubed Themis 60–300 kV operating at 200 kV (Thermo Fisher Scientific, Portugal) for 5 nm TiO_2_NPs. The colloidal stability was characterized by dynamic light scattering and zeta potential using the SZ-100 device (Horiba, ABX SAS, Amadora, Portugal). The nano crystallite size of 5 nm TiO_2_NPs was estimated by XRD pattern using the Scherrer equation. XRD pattern was collected on a X’Pert PRO diffractometer (PANalytical) set at 45 kV and 40 mA, using Cu Kα radiation (λ = 1.541874 Å) and a PIXcel detector.

Scanning electron microscopy (SEM) images for the 5 nm TiO_2_NPs localization and identification on seaweed surface were obtained with a FEI Quanta 650 FEG SEM in combination with Energy Dispersive X-Ray analysis (EDX), with an Everhart-Thornley secondary electron (ETD) operating at high vacuum, at an acceleration voltage of 10 kV and spot size of 3.0 (FEI Europe B.V.). SEM images for 25 nm TiO_2_NPs were acquired using a Helios G2 NanoLab 450S dual-beam focused ion beam SEM in combination with EDX, with an Everhart-Thornley secondary electron (ETD) operating at high vacuum, at an acceleration voltage of 10 kV (FEI Europe B.V). The obtained data from EDX were treated using Origin 9.0. The SEM samples were coated with conductive carbon using an EM ACE600 coating system (Leica microsystems).

The analysis of the internalization of NPs and their identification was carried out by high-resolution TEM (HRTEM) and HR scanning TEM (HRSTEM) coupled with EDX using a Probe-Corrected FEI Titan G2 ChemiSTEM TEM operating at an acceleration voltage of 80 kV for 25 nm TiO_2_NPs exposed samples and FEI Titan (G3) Cubed Themis 60–300 kV, operating at 60 Kv for 5 nm TiO_2_NPs exposed samples. The TEM samples were prepared using an EM TP Tissue processor (Leica microsystems) and the ultrathin sections (70–80 nm thick) were prepared using a PowerTome PC ultramicrotome (RMC Boeckeler, USA).

### Reagents

Commercially available 25 nm TiO_2_NPs and 5 nm TiO_2_NPs powders were supplied by Sigma-Aldrich (Merk Life science, product code: 718467; 99.5% purity, with a mixture of rutile and anatase) and Nanostructured and Amorphous Materials, Inc. (Katy, TX, USA; anatase, 5 nm size, 99%) respectively, and used without any further purification. The citrate NPs stock dispersions were prepared in ultrapure water (see electronic supplementary information).

Ultrapure water (18 MΩ cm of resistivity), obtained from a Milli-Q^®^ IQ7003 (Millipore Co., Bedford, MA, USA) purification system, was used as the main solvent to prepare solutions. A titanium ionic standard of 1000 mg L^−1^ H_2_O/0.24% F^-^ (PerkinElmer, Shelton, USA) was employed to prepare the calibration standards for ICP-MS and SP-ICP-MS measurements. For microwave-assisted acid digestion, 69% (w/v) nitric acid (SUPRAPUR^®^, Sigma Aldrich, Darmstadt, Germany) and 33% (w/v) hydrogen peroxide (ACS, ISO, AppliChem Panreac, Barcelona, Spain) were used. Tetramethylammonium hydroxide (TMAH) 25% (v/v) solution in water (Merck, Darmstadt Germany) was used for the basic ultrasound-assisted extraction. Glycerol for analysis (ACS, EMSURE^®^, Merck, Darmstadt, Germany) was used for dilution of the TMAH extracts before SP-ICP-MS analysis. Gold ionic standard of 1000 mg L^−1^ in 2 mol L^−1^ HCl (Merck, Darmstadt, Germany) and a suspension of Gold Nanospheres of 50 nm (49.6 nm by TEM) and 9.89 × 10^6^ particles mL^−1^ (NanoComposix, San Diego, California, USA) in aqueous 1 mM citrate were used to calculate the transport efficiency in SP-ICP-MS. Argon and ammonia (both with 99.999% of purity) from Nippon Gases (Madrid, Spain) were required for ICP-MS operation.

To prepare the samples for TEM analysis, paraformaldehyde, sodium cacodylate trihydrate (Sigma-Aldrich, Merck Life Science, Algés, Portugal); glutaraldehyde (25% in water, specially purified for use as an electron microscopy fixative; Agar Scientific, Dias de Sousa S.A, Alcochete, Portugal); propylene oxide (ReagentPlus® ≥ 99%; Sigma-Aldrich, Merck Life Science, Algés, Portugal); osmium tetroxide (2% and 4% aqueous solution), epoxy resin (EMBed-812 kit) (Science Services, Munich, Germany); and ethanol were used for the fixation, dehydration, and resin embedding of the tissue.

### Seaweed sample cultivation and exposure to TiO_2_NPs

The titanium dioxide nanoparticles bioaccumulation assays were performed in two kinds of edible seaweed species, *Palmaria palmata* (Dulse) and *Ulva* sp. (Sea lettuce). Besides, two sizes of TiO_2_NPs (25 and 5 nm TiO_2_NPs, both citrate-coated) and two different exposure doses (nominal concentrations of 0.1 and 1.0 mg L^−1^ TiO_2_NPs) were evaluated in the study. In total, four trials were carried out at different times. These bioaccumulation trials consisted of (1) *Palmaria palmata* exposed to 25 nm TiO_2_NPs, (2) *Palmaria palmata* exposed to 5 nm TiO_2_NPs, (3) *Ulva* sp. exposed to 25 nm TiO_2_NPs, and (4) *Ulva* sp. exposed to 5 nm TiO_2_NPs.

Details about seaweed sample cultivation and exposure to TiO_2_ NPs are included in the electronic supplementary information. Three seaweed sample replicates were collected from each tank on days 0, 7, 14, 21, and 28 for analysis by ICP-MS, TEM, and SEM.

Seaweed sample preparation for SEM and TEM analysis was carried out after 28 days of exposure to TiO_2_NPs. Two seaweed samples of each specie from each treatment group were used to collect 0.7 cm square fragments of seaweed leaf. The fragments were fixated overnight at 4 °C in Karnovsky fixative solution, which involves a mixture of 2% paraformaldehyde and 2.5% glutaraldehyde in 0.1 M sodium cacodylate buffer. These fragments were stored at 4 °C under shaking for later use. The bigger fragments of the seaweed (around 2 cm squares) were fixated in 10% neutral buffered formalin for 48 h (replacing the formalin solution every 24) and subsequently stored in 70% ethanol at room temperature until further use. The SEM and TEM fragments were stored until their processing routine for electron microscopy (EM) analysis.

### Microwave acid-assisted digestion

A microwave-assisted acid digestion method is necessary to destroy the seaweed matrix for total Ti determination by ICP-MS. Seaweed samples were digested with a mixture of nitric acid and hydrogen peroxide following the method described in a previous work [28]. The digested samples were diluted with ultrapure water (dilutions 1:10 to 1:100) until 25 mL before their analysis by ICP-MS. This procedure was applied in triplicate to each sample, and two blanks were prepared for each set of experiments.

### TMAH ultrasound-assisted TiO_2_NPs extraction

A basic extraction with 2.5% tetramethylammonium (TMAH) was selected to isolate TiO_2_NPs from the seaweed matrix without changing the primary size and concentration of the metal particles. This procedure was developed in a previous study carried out in our laboratory [28], at it was applied in triplicate to each sample; two blanks were prepared for each set of operational conditions or day of analysis. Finally, the extracts were diluted with 1% (v/v) glycerol before SP-ICP-MS analysis (dilutions from 1:100 to 1:6000).

### Total titanium determination by ICP-MS and TiO_2_NPs analysis by SP-ICP-MS

Total titanium concentration in the digested samples was determined by the ICP-MS, using the conditions listed in Table S1 (electronic supplementary information). The dynamic reaction cell (DRC), with ammonia as reaction gas (flow rate 1.0 mL min^−1^), was used to minimize interferences in the determination of titanium. The purpose of ammonia is to form the Ti-NH_3_ cluster [^48^Ti^14^N^1^H(^14^N^1^H_3_)_4_^+^] of *m/z* 131 [29]. Standard addition calibration with ionic Ti concentrations between 0 and 50.0 μg L^−1^ was used to correct the matrix effect.

The TiO_2_NPs content was measured in the alkaline extracts using the operational conditions of SP-ICP-MS (DRC mode) that are listed in Table S1. Transport efficiency (TE (%)) was automatically calculated by the software Syngistix™ Nano after the introduction of an ionic gold calibration from 0 to 3.0 μg L^−1^ and Au nanospheres (49.6 nm in size) at a concentration of 9.89 × 10^4^ particles mL^−1^. SP-ICP-MS measurements were performed using a titanium external calibration prepared in ultrapure water in a concentration range between 0 and 10.0 μg L^−1^. TMAH extracts were diluted with 1% (v/v) glycerol before SP-ICP-MS analysis. Finally, the TiO_2_NPs concentration and size distributions in the diluted extracts were directly obtained from the software after analysis and used to calculate the concentrations in the seaweed samples.

### Localization and identification of TiO_2_NPs on/in seaweed tissue by electron microscopy techniques

SEM coupled with EDX analysis of both fixated TiO_2_NPs exposed *Palmaria palmata* and *Ulva* sp. fragments were performed to study the interaction/association of both TiO_2_NPs, 5 and 25 nm, with the seaweed’ surface as well as their identification by EDX. The fixated fragments were cut into smaller pieces of < 10 mm square, placed on pin stubs (standard 12.7 mm, 8 mm pin length, Ted Pella), and subsequently coated with conductive carbon using an EM ACE600 coating system (Leica microsystems). The samples were analyzed using the SEM-EDX system described in Section 2.1.

The internalization and localization of both TiO_2_NPs as well as their identification were investigated via a 28-day exposure to both seaweeds by STEM-EDX. The fixated fragments were subjected to a TEM processing routine. Osmium tetroxide solution at 1% was used for the post-fixation step. The fragments were dehydrated by sequential washings in increasing solutions in ethanol until 100% and finally with propylene oxide. This was also used for gradual impregnation with the epoxy resin. The fragments were placed in silicone molds with resin and left for three days to cure at 60 °C. Ultrathin sections (≈80 nm thick) were made in an ultramicrotome (Section 2.1), with a diamond knife (Diato), and placed on formavar/carbon 200 mesh grids. TEM and STEM micrographs of the sections and the EDX mapping were acquired with the electron microscopes described in Section 2.1.

## Results and discussion

### TiO_2_NPs characterization

The initial stock dispersions of citrate-25 nm TiO_2_NPs and citrate-5 nm TiO_2_NPs were characterized by TEM, DLS, and zeta potential. Figure S1 (electronic supplementary section) shows representative TEM images of both NPs dispersed in ultrapure water at a concentration of 50 mg L^−1^. TEM images show that both NPs tend to form aggregates. The size of primary particles of the citrate-25 nm TiO_2_NPs can be estimated as 29 ± 10 nm; however, this estimation for citrate-5 nm TiO_2_NPs was not possible despite having used an aberration-corrected HRTEM because the NPs overlap each other in the aggregate. The nano crystallite size of this 5 nm TiO_2_NPs was calculated by X-ray diffraction (XRD) pattern using the Scherrer equation [30], obtaining a size of 8.6 nm (Fig. S2). The sonication of TiO_2_NPs standards was necessary to avoid the common phenomena of TiO_2_NPs aggregation.

Table S2 shows the characterization of both NPs before exposure to ultrapure water and artificial seawater at 50 mg L^−1^ of concentration, by DLS and zeta potential. The TiO_2_NPs in extracts of exposed seaweed were analyzed by SP-ICP-MS, previous dilution with ultrapure water and sonication. The NPs sizes obtained were bigger than their primary size, and smaller sizes were obtained in seaweed extracts (128 nm versus 52 nm for citrate-25 nm TiO_2_NPs, and 65 nm versus 48 nm for citrate-5 nm TiO_2_NPs). This could be attributed to the variation of the aggregation degree produced by the addition of the nanoparticles prepared in F2P growing media and mixed with phytoplankton, to the possible interaction of the algae’ polysaccharides or other components such as polyphenols [31], and subsequently the TMAH extraction and sonication previous to analysis.

The colloidal stability was also analyzed in artificial seawater since the bioaccumulation assays were performed with marine algae cultured in a medium based on seawater. Both TiO_2_NPs aggregated immediately after their dispersion in artificial seawater as shown in the DLS analysis (Table S2). The Z-potential values were almost zero in this medium for both NPs, which supports the NPs’ destabilization due to the very high ionic strength of the seawater (i.e., high concentration of salts) [32].

### Total titanium determination in seaweed samples exposed to TiO_2_NPs

The total titanium concentration was determined following the procedure described in Section 2.6. Average concentrations were calculated using the nine replicates of seaweed taken from the three tanks with the same exposure dose (3 replicates per tank and per sampling day). The limit of detection (LOD) and the limit of quantification (LOQ) were 1.5 and 4.8 ng g^−1^, respectively. The repeatability (*n* = 5) in the measurements resulted in a relative standard deviation (RSD) < 6%, and the analytical recovery percentages were 91 ± 5% and 113 ± 5%, respectively, for 0.5 and 10 μg L^-1^ of titanium added (*n* = 5).

#### Variation of total titanium content in *Palmaria palmata* (red seaweed)

**Fig. 1:**
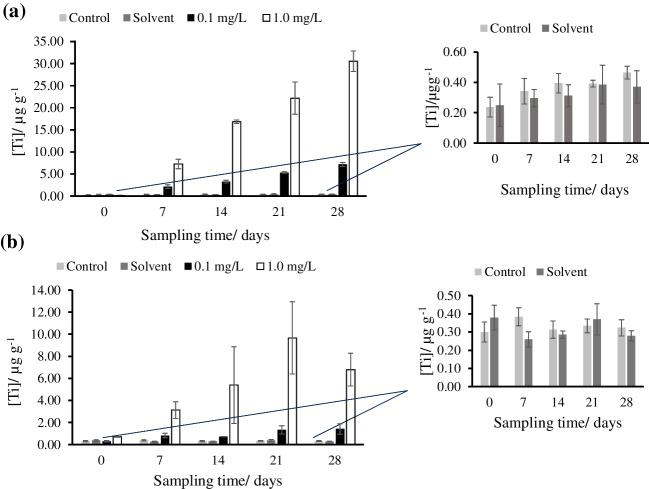
Total Ti bioaccumulation in *Palmaria palmata* exposed to **a** 25 nm TiO_2_NPs and **b** 5 nm TiO_2_NPs for 28 days

Titanium was determined in *Palmaria palmata* exposed to different concentrations of TiO_2_NPs of two different sizes (25 and 5 nm) for 28 days by ICP-MS. Figure 1 shows the mean concentrations and standard deviations of titanium in *Palmaria palmata* exposed to these NPs. Table S3 shows the ranges of total titanium content (0–28 days) in *Palmaria palmata* for control, solvent, low exposure dose (0.1 mg L^-1^ of 25 nm TiO_2_NPs or 5 nm TiO_2_NPs), and high exposure dose (1.0 mg L^-1^ of 25 nm TiO_2_NPs or 5 nm TiO_2_NPs) tanks.

These results showed that titanium bioaccumulation in *Palmaria palmata* exposed to 25 nm TiO_2_NPs, at a high dose of exposure, increased with time until 28 days. Lower bioaccumulation was observed for *Palmaria palmata* exposed to 5 nm TiO_2_NPs, and the results showed greater variability. As can be observed, titanium concentration increased with the sampling time until 21 days, for seaweeds exposed to 1.0 mg L^-1^ of these NPs, and then decreased.

Figure S3 shows in detail the total titanium concentrations measured in seaweed grown in control (S3.a), solvent (S3.b), 0.1 mg L^-1^ 25 nmTiO_2_NPs (S3.c), and 1.0 mg L^-1^ 25 nm TiO_2_NPs (S3.d) media for 28 days. As can be observed in the figure, titanium concentration in control tanks and in citrate-containing tanks remained approximately constant with exposure time (from 0 to 28 days). In both cases, the *P*-values obtained after the application of a one-way ANOVA test were greater than 0.05 (software Statgraphics XVIII, Warrenton, USA); then, no statistically significant differences were observed between the averages from the different sampling days at a 5% of the significance level. The concentrations of titanium in seaweed grown in tanks exposed to 0.1 mg L^-1^ and 1.0 mg L^-1^ 25 nm TiO_2_NPs increased with the exposure time up to 7.15 ± 0.43 μg g^-1^, and 30.55±2.31 μg g^-1^ (both at day 28; values mean of three tanks), respectively. The accumulation was dose-dependent, and the concentration measured was 4.3 higher after exposure to 1.0 mg L^-1^ 25 nm TiO_2_NPs. There were significant variations in the average concentrations of titanium (*P*-value < 0.05). The multiple range test confirmed a linear increase in the concentration of titanium with time in seaweed exposed to 0.1 mg L^-1^, but in the case of seaweed exposed to 1.0 mg L^-1^, there was an increase in the concentration till day 14, and no significant differences between the concentrations were determined in days 14 and 21.

Figure S4 shows in detail the total titanium concentration measured in seaweed grown in control (S4.a), solvent (S4.b), 0.1 mg L^-1^ of 5 nm TiO_2_NPs (S4.c), and 1.0 mg L^-1^ of 5 nm TiO_2_NPs (S4.d) media. The titanium concentration in control and solvent tanks remained constant with exposure time, and no significant differences were found between the average concentrations at the sampling days (in both cases *P*-value was higher than 0.05). Titanium concentrations in seaweed exposed to 0.1 mg L^-1^ and 1.0 mg L^-1^ of 5 nm TiO_2_NPs increased with the exposure time up to 1.42 ± 0.48 μg g^-1^ and 6.78 ± 1.48 μg g^-1^ (both at day 28), respectively. The ANOVA tests showed significant differences in concentrations between the sampling days (*P*-value < 0.05) for *Palmaria palmata* exposed to 0.1 and 1.0 mg L^-1^. However, the multiple range test did not show significative differences between 7, 14, 21, and 28 days, and between 14, 21, and 28 days for *Palmaria palmata* exposed to 0.1 and 1.0 mg L^-1^ of 5 nm TiO_2_NPs, respectively.

#### Variation of total titanium content in *Ulva* sp. (green seaweed)

**Fig. 2:**
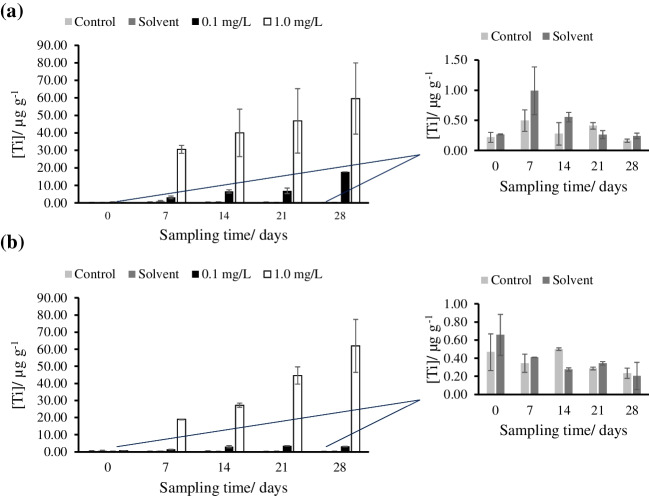
Total Ti bioaccumulation in *Ulva* sp. exposed to **a** 25 nm TiO_2_NPs and **b** 5 nm TiO_2_NPs for 28 days

Titanium was determined in *Ulva* sp. exposed to different concentrations of the element present as TiO_2_NPs nanoparticles of two different sizes (25 and 5 nm) by ICP-MS. Figure 2 shows the mean concentration of titanium measured in the tanks and the total titanium bioaccumulation pattern in *Ulva* sp*.* exposed to (a) 25 nm and (b) 5 nm TiO_2_NPs. Total titanium concentration ranges (0–28 days) in *Ulva* sp. for control, solvent, low exposure dose (0.1 mg L^-1^ of 25 nm TiO_2_NPs or 5nm TiO_2_NPs), and high exposure dose (1.0 mg L^-1^ of 25 nm TiO_2_NPs or 5 nm TiO_2_NPs) tanks are shown in Table S3. Figure S5 also shows the total titanium concentration in seaweed grown in control (S5.a), solvent (S5.b), 0.1 mg L^-1^ of 25 nm TiO_2_NPs (S5.c), and 1.0 mg L^-1^ of 25 nm TiO_2_NPs (S5.d) media.

In the case of *Ulva* sp., the control tanks and the solvent tanks had greater variability than for *Palmaria palmata* where the concentrations remained practically constant. The total titanium bioaccumulation in *Ulva* sp. at a high exposure dose increased with the exposure time until 28 days in both experiments.

For *Ulva* sp. exposed to 25 nm TiO_2_NPs, the basal titanium concentration remained constant with exposure time from 0 to 28 days. The ANOVA test showed no significant difference in concentrations with time (*P*-value > 0.05). The concentration in tanks exposed to citrate coating also remained constant with exposure time, but there was an increase in concentration on day 7 in the three tanks, after which the concentration decreased. The concentration on the 7th day was statistically different from the other average concentrations at different sampling days, as observed after applying an ANOVA test (*P*-value < 0.05) and the multiple range test, but no statistical differences were found between 0, 14, 21, and 28 days (multiple range test). The concentrations in seaweed from tanks exposed to 0.1 mg L^-1^ 25 nm TiO_2_NPs were not statistically significative different from the 7–28 days exposure period (*P*-value > 0.05). The concentrations in tanks exposed to 1.0 mg L^-1^ of 25 nm TiO_2_NPs showed a linear increasing trend with the exposure time up to 59.63 ± 20.3 μg g^-1^ (at day 28, mean of the three tanks J-L). This concentration was approximately nine times higher than that measured after exposure at 0.1 mg L^-1^ of 25 nm TiO_2_NPs. The ANOVA test showed that there is a significant difference in concentration with exposure time (*P*-value < 0.05). Furthermore, the multiple range test showed the overlapping in concentration intervals at 7, 14, 21, and 28 days, with differences between days 0 and 7, 14, 21, and 28 days and differences between 7 and 28 exposure days.

Figure S6 shows the total titanium concentration in seaweed grown in control (S6.a), solvent (S6.b), 0.1 mg L^-1^ of 5 nm TiO_2_NPs (S6.c), and 1.0 mg L^-1^ of 5 nm TiO_2_NPs (S6.d) tanks. For *Ulva* sp. exposed to 5 nm TiO_2_NPs, the titanium concentration in control and solvent tanks remained constant with exposure time. The concentration measured was slightly higher at day 0 in the tanks. But in both cases, the ANOVA test and the multiple range tests showed no significant difference (*P* > 0.05) among the tanks from day 7 to day 28. The concentrations in seaweed from tanks exposed to 0.1 mg L^-1^ of 5 nm TiO_2_NPs increased with the exposure time until 14 days, and stabilized after that, reaching a concentration of 3.06 ± 0.18 μg g^-1^ on the 28th day. The ANOVA test for *Ulva* sp. exposed to 0.1 mg L^-1^ showed a statistical difference (*P* < 0.05) among the average concentrations as a function of the sampling time, and the multiple range test showed an overlapping in the concentrations after 14 days. The total Ti concentrations in seaweed from tanks exposed to 1.0 mg L^-1^ of 5 nm TiO_2_NPs had a linear increasing trend with exposure time until 61.96 ± 15.49 μg g^-1^ at day 28 (mean of the three tanks J-L; concentration 20 times higher than after exposure to 0.1 mg L^-1^ of 5 nm TiO_2_NPs). The ANOVA test for *Ulva* sp. exposed to 1.0 mg L^-1^ showed a statistical difference (*P* < 0.05) among the average concentrations as a function of the sampling time. The multiple range test confirmed the overlapping of titanium concentrations at 14 and 21 days and afterward an increase at day 28.

In summary, the bioaccumulation of total Ti in *Palmaria palmata* exposed to 25 nm TiO_2_NPs was lower than for *Ulva* sp. exposed to 25 nm TiO_2_NPs. *Palmaria palmata* reached 30.55 ± 2.31 μg g^-1^ and *Ulva* sp. reached 59.63 ± 20.3 μg g^-1^ of total titanium. However, the bioaccumulation in *Palmaria palmata* was more gradual and the reproducibility was better than for *Ulva* sp. (which already exhibited a sharp increase in Ti concentration on day 7). The bioaccumulation in *Palmaria palmata* exposed to 5 nm TiO_2_NPs was also lower than for *Ulva* sp. exposed to 5 nm TiO_2_NPs. *Ulva* sp. reached a concentration of titanium of 61.96 ± 15.49 μg g^-1^ whereas the maximum *Palmaria palmata* concentration was 8.08 ± 2.50 μg g^-1^ (on the 21st day). This different behavior between species is normal, and it can be attributed to the various chemical composition of seaweed, e.g., to the different polysaccharides present in cell walls [33]. The different assimilation of TiO_2_NPs in different tanks containing the same seaweed could be due to small differences between the tanks (e.g., differences in light, water flow, feed).

This study corroborates the use of *Ulva* sp. as one of the most important macroalgae species used for monitoring seawater pollution, with a big surface area and elevated capacity to bioaccumulate metals, and also applied to evaluate the presence of titanium on the coast of South Africa during the different seasons [20]. Desideri et al. [23] measured concentrations in edible seaweed purchased in Italian supermarkets. The concentration of Ti ranged from 1.7 ± 0.1 (*Ulva enteromorpha*) to 156 ± 10.9 (*Ulva lactuca*) mg kg^-1^ d.w. in *Ulva* sp., while the concentration of *Palmaria palmata* was 5.3 ± 0.4 mg kg^-1^. The highest concentration of Ti was found by this research group in *Lithothamnium calcareum* (1364 ± 95.5 mg Kg^-1^) [23].

### Analysis of TiO_2_NPs in seaweed

The TiO_2_NPs g^-1^ content and the size distribution of the nanoparticles in seaweed were determined by SP-ICP-MS following the procedure described in Section 2.6. The experimental TE (%) of the instrument was approximately 9 ± 1% for flow rates between 0.189 and 0.210 mL min^-1^. The LOD and LOQ were calculated using the Laborda et al. criteria [34]. The LOD_number_ were 2.82 × 10^6^ TiO_2_NPs L^-1^ (instrumental LOD) and 2.82 × 10^6^ TiO_2_NPs g^-1^ (referred to sample, for 1:100 dilution). The LOD_size_ were 27 and 23 nm for 5σ and 3σ criteria, respectively. The 5σ criteria was used to avoid the presence of false positives. Averages were taken for the nine replicates of seaweed for each dose of exposure on each sampling day. The size analysis by DLS and TEM (described in Section 3.1.) confirmed that both kinds of particles (25 nm TiO_2_NPs and 5 nm TiO_2_NPs) formed aggregates that were larger than LOD_size_ (> 27 nm) both before and after nanoparticle exposure. This typical aggregation in TiO_2_NPs allowed performing the characterization of NPs by SP-ICP-MS.

**Fig. 3:**
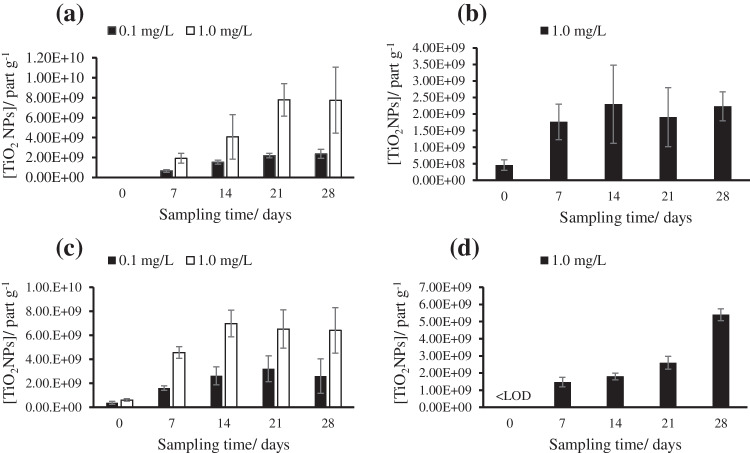
TiO_2_NPs bioaccumulation in *Palmaria palmata* exposed to **a** 25 nm TiO_2_NPs and **b** 5 nm TiO_2_NPs, and in *Ulva* sp. exposed to **c** 25 nm TiO_2_NPs and **d** 5 nm TiO_2_NPs

As total titanium analysis confirmed the absence of bioaccumulation of 25 nm TiO_2_NPs and 5 nm TiO_2_NPs in the control and solvent tanks, the alkaline TMAH extractions were performed only in the tanks with seaweed exposed to low and high exposure doses for both sizes of particles. Figure 3 shows the mean concentrations of nanoparticles measured in the extracts after exposure of 25 nm TiO_2_NPs (at 0.1 and 1.0 mg L^-1^ exposure doses) and 5 nm TiO_2_NPs (at 1.0 mg L^-1^ exposure dose) for *Palmaria palmata* and *Ulva* sp. Table S4 shows the concentration of 25 nm TiO_2_NPs and 5 nm TiO_2_NPs, the most frequent sizes, and mean sizes range (0–28 days), at high and low exposure doses in the extracts of samples. As it can be observed, in the case of 5 nm TiO_2_NPs, the tanks with seaweed exposed to a dose of 0.1 mg L^-1^ resulted in nanoparticle concentrations lower than LOD_number_ (< 2.82 × 10^6^ TiO_2_NPs g^-1^).

#### Size distribution and TiO_2_NPs content in *Palmaria palmata*

**Fig. 4:**
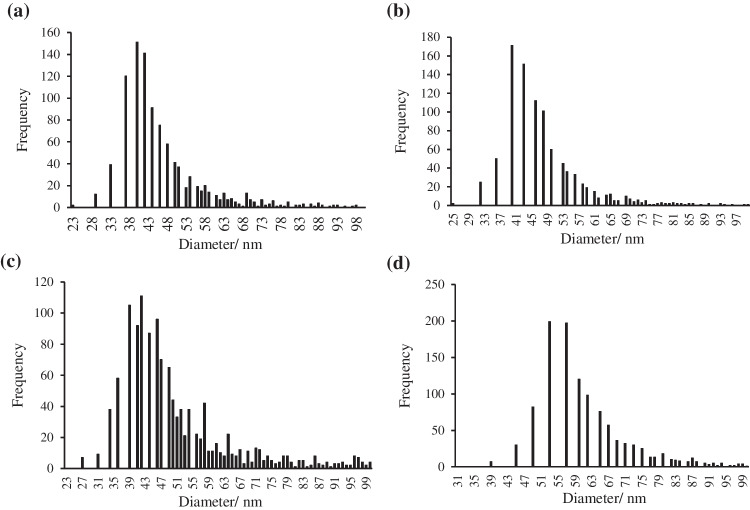
Size distribution histograms of TiO_2_NPs corresponding to extracts of *Palmaria palmata* exposed to **a** 0.1 mg L^-1^ 25 nm TiO_2_NPs and **b** 1.0 mg L^-1^ 5 nm TiO_2_NPs, and *Ulva* sp. exposed to **c** 0.1 mg L^-1^ 25 nm TiO_2_NPs and **d** 1.0 mg L^-1^ 5 nm TiO_2_NPs

Nanoparticle content and size distributions were studied in samples of alkaline extracts of *Palmaria palmata* exposed to 25 and 5 nm TiO_2_NPs. Nanoparticle size distribution histograms of *Palmaria palmata* and *Ulva* sp. exposed to 0.1 mg L^-1^ of 25 nm TiO_2_NPs or 1.0 mg L^-1^ of 5 nm TiO_2_NPs are shown in Fig. 4. Figure S7 shows *Palmaria palmata* nanoparticle content in seaweed from tanks exposed to (a) 0.1 mg L^-1^, (b) 1.0 mg L^-1^ of 25 nm TiO_2_NPs, and (c) 1.0 mg L^-1^ of 5 nm TiO_2_NPs. Figure S8 shows the most frequent and mean sizes of *Palmaria palmata* exposed to (a) 0.1 mg L^-1^, (b) 1.0 mg L^-1^ of 25 nm TiO_2_NPs, and (c) 1.0 mg L^-1^ of 5 nm TiO_2_NPs.

For *Palmaria palmata* exposed to 25 nm TiO_2_NPs, the TiO_2_NPs content in tanks exposed to 0.1 mg L^-1^ of NPs increased linearly with the exposure time until 2.37 × 10^9^ ± 4.49 × 10^8^ NPs g^-1^ (day 28). The TiO_2_NPs content in tanks exposed to 1.0 mg L^-1^ showed bioaccumulation with exposure time until 7.75 × 10^9^ ± 3.31 × 10^9^ NP g^-1^ (day 28; concentration 3.3 times higher than after exposure to 0.1 mg L^-1^). For *Palmaria palmata* exposed to 0.1 mg L^-1^ 25 nm TiO_2_NPs, the most frequent sizes (mean of three tanks) were between 40 ± 2 nm (at day seven) and 41 ± 1 nm (at day 28), and the mean sizes were between 52 ± 2 nm (day 7) and 54 ± 2 nm (day 28). However, for *Palmaria palmata* exposed to 1.0 mg L^-1^ 25 nm TiO_2_NPs, the most frequent sizes were between 49 ± 6 nm (day 7) and 47 ± 3 nm (day 28), and the mean sizes were 70 ± 9 nm (days 7) and 64 ± 6 nm (day 28). It seems that TiO_2_NPs tend to aggregate with increasing concentration, but the sizes remained constant at the same doses with exposure time.

For *Palmaria palmata* exposed to 5 nm TiO_2_NPs, the TiO_2_NPs content in tanks exposed to 0.1 mg L^-1^ of NPs was under the LOD_number_ (< 2.82 × 10^6^ NPs L^-1^). In the case of high-dose exposed tanks, the NPs content reached 2.23 × 10^9^ ± 4.39 × 10^8^ NPs g^-1^ (day 28). The most frequent sizes varied from 33 ± 1 nm (day 0) to 41 ± 1 nm (day 28), and the mean sizes varied from 38 ± 1 nm (day 0) to 50 ± 1 nm (day 28).

**Fig. 5:**
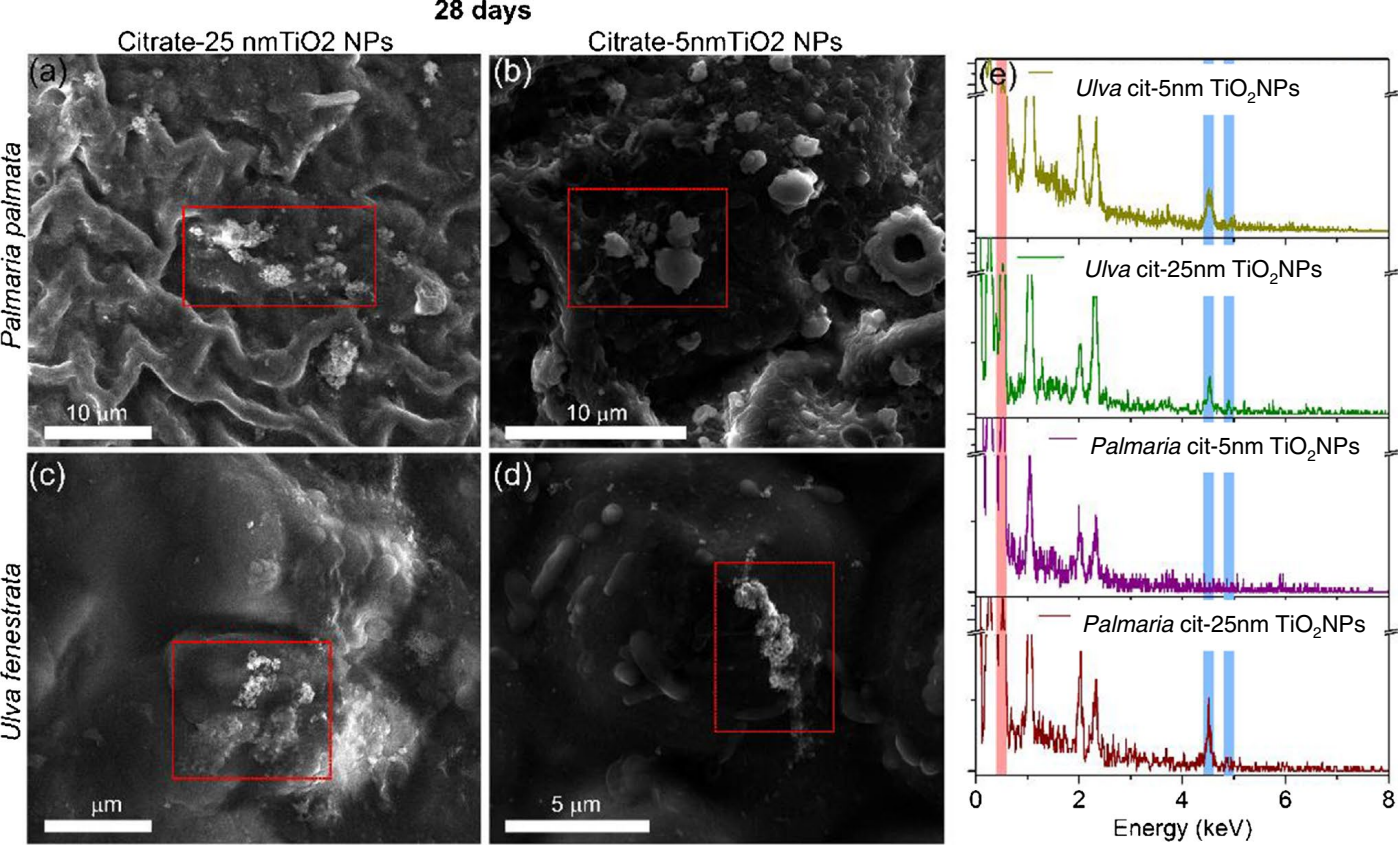
Representative SEM images of **a**, **b** *Palmaria palmata* and **c**, **d** *Ulva* sp. exposed for 28 days to 1.0 mg L^-1^ of **a**, **c** 25 nm TiO_2_NPs and **b**, **d** 5 nm TiO_2_NPs. **e** It shows the corresponding EDX spectra acquired in the limited area indicated by a dashed red square. The shadowed areas indicate the peaks corresponding to oxygen (pink) and titanium (blue) elements

SEM, TEM, and STEM techniques were employed to examine the localization of the NPs and EDX analysis to identify the NPs found. Figure 5a and b shows SEM images of *Palmaria palmata* exposed for 28 days to 1.0 mg L^-1^ of both NPs sizes, respectively. Aggregates with a size bigger than 1 μm were found, and they were identified as titanium dioxide in the sample exposed to 25 nm TiO_2_NPs. However, none of the aggregates could be identified as TiO_2_ in the case of samples exposed to 5 nm TiO_2_NPs. In both cases, no NP was observed/localized into the tissues by TEM or STEM micrographs (data not shown) after 28 exposure days, and therefore, the internalization could not be confirmed by electron microscopy. The bioaccumulation of 5 nm TiO_2_NPs in *Palmaria palmata* measured as total titanium content was lower than for 25 nm TiO_2_NPs; this could explain why it was not possible to localize the aggregated NPs using SEM or TEM. However, more information could be extracted from electron microscopy analysis for citrate-25 nm TiO_2_NPs. It seems that aggregates bigger than 1 μm were associated with the seaweed surface most likely by adsorption as reported previously. The adsorption capacity of TiO_2_NPs by algae was in the range of 59 to 5701 μg g^-1^ d.w. (dry weight) in 72 h [35]. The initial concentration and the colloidal stability of the NPs affect the adsorption kinetic. In this case, the NPs dispersed in the medium formed bigger aggregates than in ultrapure water as demonstrated by DLS analysis (Table S2). Therefore, it is expected that the aggregates would adsorb on the surface instead of being internalized due to their size. It should be highlighted that the size of the aggregates is different in SEM analysis when compared to the one obtained by SP-ICP-MS. This could be attributed to a stabilization effect of phytoplankton during seaweed exposure or in the extraction procedure due to the presence of polysaccharides and polyphenols from the seaweed, which could coat the NPs forming smaller aggregates.

#### Size distribution and TiO_2_NPs content in *Ulva* sp.

Nanoparticle content and size distributions were studied in alkaline extracts from *Ulva* sp. exposed to 25 and 5 nm TiO_2_NPs. Figure S9 shows the *Ulva* sp. nanoparticles content in tanks with seaweed exposed to (a) 0.1 mg L^-1^, (b) 1.0 mg L^-1^ of 25 nm TiO_2_NPs, and (c) 1.0 mg L^-1^ of 5 nm TiO_2_NPs. Figure S10 shows the most frequent and mean sizes of *Ulva* sp. exposed to (a) 0.1 mg L^-1^, (b) 1.0 mg L^-1^ of 25 nm TiO_2_NPs, and (c) 1.0 mg L^-1^ of 5 nm TiO_2_NPs.

For *Ulva* sp. exposed to 25 nm TiO_2_NPs, the TiO_2_NPs content in tanks exposed to 0.1 mg L^-1^ of NPs increased with exposure time until 2.60 × 10^9^ ± 1.44 × 10^9^ NPs g^-1^ (day 28; concentration 2.5 times higher than after exposure to 0.1 mg L^-1^). The TiO_2_NPs content in tanks exposed to 1.0 mg L^-1^ showed a stabilized bioaccumulation until 6.41 × 10^9^ ± 1.90 × 10^9^NPs g^-1^ (day 28). For *Ulva* sp. exposed to 0.1 mg L^-1^ 25 nm TiO_2_NPs, the most frequent sizes were between 36 ± 2 nm (day 0) and 43 ± 6 nm (day 28), and the mean sizes were between 52 ± 2 nm (day 0) and 54 ± 5 nm (day 28). However, for *Ulva* sp. exposed to 1.0 mg L^-1^ of 25 nm TiO_2_NPs, the most frequent sizes varied from 44 ± 7 nm (day 0) to 52 ± 14 nm (day 28), and the mean sizes from 53 ± 9 nm (day 0) to 66 ± 15 nm (day 28). Like in *Palmaria palmata*, TiO_2_NPs tend to aggregate with increasing concentration, but the sizes remained constant at the same doses with the exposure time. This could explain the increase in size measured in SP-ICP-MS. Other authors have observed the increase in the size of nanoparticles measured in plant extracts after exposure in comparison to the primary particles, e.g., in the case of AgNPs [36]. However, Deng et al. [37] did not find significant differences between the most frequent and the mean sizes of titanium dioxide NPs or BPs (bulk particles) measured by SP-ICP-MS in nanoparticle controls and after the enzymatic or acid extraction of TiO_2_NPs from rice.

For *Ulva* sp. exposed to 5 nm TiO_2_NPs the content of nanoparticles in tanks exposed to 0.1 mg L^-1^ of NPs was under the LOD_number_ (< 2.82 × 10^6^ TiO_2_NPs g^-1^). However, for 1.0 mg L^-1^ of exposure dose, the bioaccumulation in seaweed increased linearly with the exposure time until 5.41 × 10^9^ ± 3.47 × 10^8^ NPs g^-1^ at 28 days. Besides, the most frequent sizes were 48 ± 3 nm (day 0) and 52 ± 3 nm (day 28), and the mean sizes were between 76 ± 4 nm (day 0) and 64 ± 1 nm (day 28). The most frequent and mean sizes remained approximately constant with the exposure time.

Although the total concentration of titanium bioaccumulated for 25 nm TiO_2_NPs was higher in *Ulva* sp. than in *Palmaria palmata* (Section 3.2), there were no significant differences in the concentrations of TiO_2_NPs bioaccumulated in both kinds of seaweed at low and high exposure 25 nm TiO_2_NPs doses. Thus, *Ulva* sp. seems to have accumulated a higher proportion of ionic titanium or contains TiO_2_NPs smaller than the LOD in size (27 nm for 5σ criteria). Besides, no differences were found in the most frequent and mean sizes in *Palmaria palmata* and *Ulva* sp. exposed to 25 nm TiO_2_NPs. In the case of 5 nm TiO_2_NPs, the total titanium bioaccumulation was also higher for *Ulva* sp. than for *Palmaria palmata*. Nevertheless, no significant differences in the TiO_2_NPs content were found in both kinds of seaweed.

**Fig. 6:**
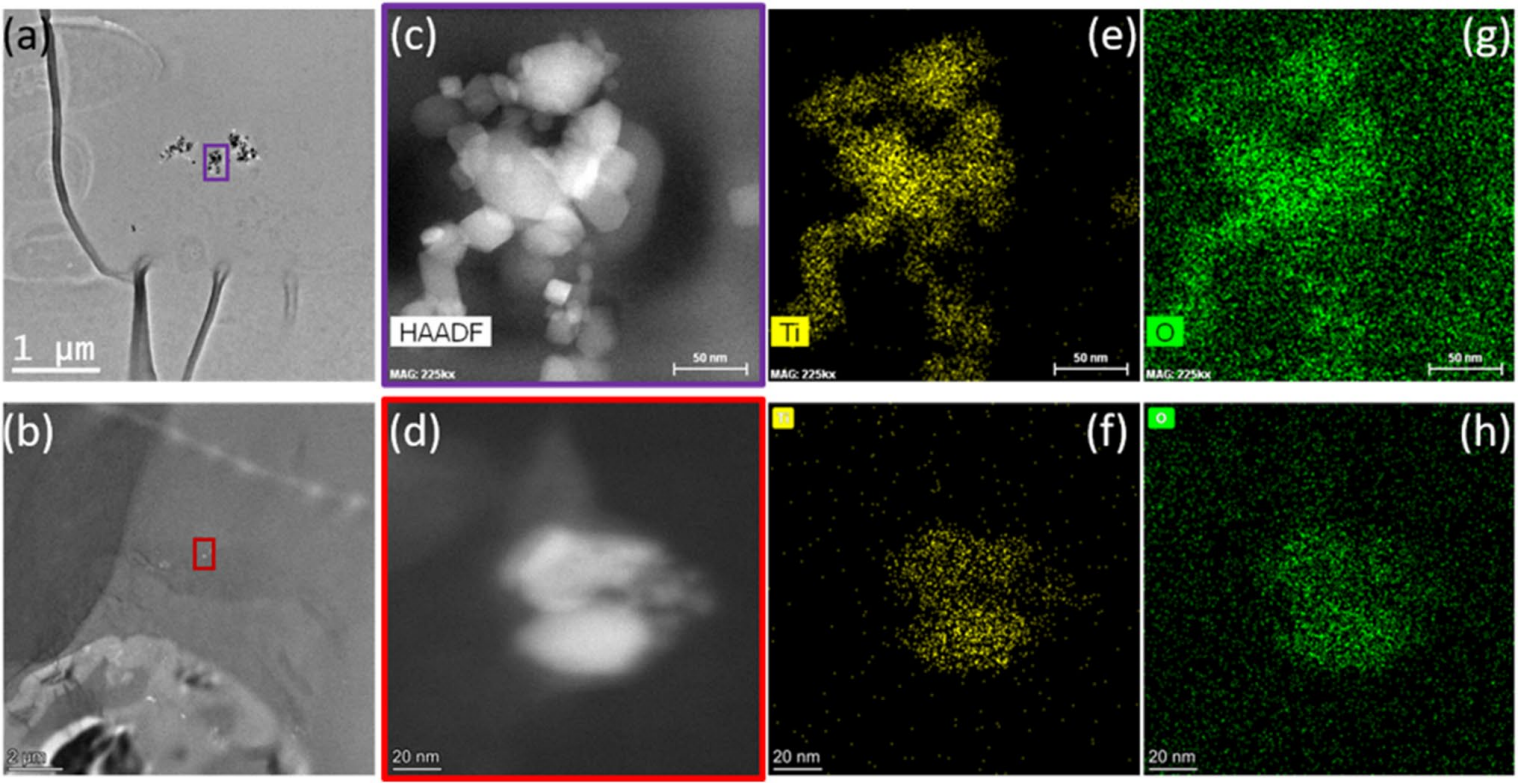
TEM micrograph of *Ulva* sp. after 28 days exposure to 1.0 mg L^-1^ of 25 nm TiO_2_NPs **a** and STEM micrograph of *Ulva* sp. after 28 days-exposed to 1.0 mg L^-1^ of 5 nm TiO_2_NPs **b**. In both cases, inorganic nanoparticles were localized. STEM-EDX maps, with the HAADF-STEM image of 25 nm TiO_2_NPs **c** and 5 nm TiO_2_NPs **d** and EDX elemental maps of Ti **e**, **f** and O **g**, **h** confirmed that the NPs localized were TiO_2_

The set of electron microscopy techniques and EDX analysis described in Section 2.1 were also employed to examine the localization and identification of the NPs in *Ulva* sp. SEM images (Fig. 5c and d) clearly show the presence of TiO_2_ aggregates (size > 1 μm) confirmed by EDX (Fig. 5e) on their surface after 28 days of exposure to both sizes of NPs studied here. In addition, the internalization could be visualized by TEM/STEM, and the identity of NPs was confirmed as titanium dioxide by EDX analysis (Fig. 6). Interestingly, the size of the internalized aggregates was smaller than the aggregates localized on the surface. The aggregates of 25 nm TiO_2_NPs presented high polydispersity (i.e., high variability in the size distribution), while fewer aggregates of 5 nm TiO_2_NPs were localized and the size was smaller than in the case of 25 nm NPs. This support the hypothesis that big aggregates were adsorbed firstly on the seaweed’s surface via interaction with the glycoproteins and polysaccharides present in the cell wall [35]. These aggregates were “broken” during the internalization forming smaller aggregates embedded in the cell wall. Thus, the interaction of the NPs with some seaweed components together with the effect of ultrasonication used during the extraction process could explain the smaller size obtained by SP-ICP-MS, as it has been reported that the use of ultrasound could improve the dispersion of TiO_2_NPs [38].

## Conclusions

The present research provides information about the bioaccumulation of TiO_2_NPs in marine macroalgae after exposure periods longer than those usually employed in other studies. The analysis of total Ti, the TiO_2_NPs content, and TiO_2_NPs size has been carried out by ICP-MS and SP-ICP-MS while the NPs localization in the tissues was investigated by SEM and TEM. Titanium concentrations measured in *Ulva* sp. were higher than those found in *Palmaria palmata* for the same conditions of exposure, and internalization was only verified in the former. Finally, the ability to bioaccumulate TiO_2_NPs of different sizes has been demonstrated in the seaweed species, resulting in more Ti ionic or small-size nanoparticulate TiO_2_ bioaccumulation in green rather than red seaweed. Due to the lack of cut-off values in the regulatory landscape, further research is needed to ensure a good level of quality and safety in alimentary products.

## Supplementary information

ESM 1
