# Supplementary material for: Bioaccumulation of titanium dioxide nanoparticles in green (Ulva sp.) and red (Palmaria palmata) seaweed
Source: Mikrochim Acta. 2023 Jul 7;190(8):287. doi: 10.1007/s00604-023-05849-1 (PMC10329078; doi:10.1007/s00604-023-05849-1)
Supplement: Supplementary file 1 — ESM 1 [file 604_2023_5849_MOESM1_ESM.docx]

**Electronic Supplementary Material for:**

**Bioaccumulation of titanium dioxide nanoparticles in green (Ulva sp.) and red (Palmaria palmata) seaweed**

**Juan José López-Mayán^1^, Blanca Álvarez-Fernández^1^, Elena Peña-Vázquez^1^, María Carmen Barciela-Alonso^1^, Antonio Moreda Piñeiro^1^, Julie Maguire^2^, Mick Mackey^2^, Monica Quarato^3^, Ivone Pinheiro^3^, Begoña Espiña^3^, Laura Rodríguez-Lorenzo^3^, Pilar Bermejo-Barrera^1,^ ***

^1^ Trace Element, Spectroscopy and Speciation Group (GETEE), Institute of Materials (iMATUS), Faculty of Chemistry, University of Santiago de Compostela, 15782 Santiago de Compostela, Spain. ^2^ Indigo Rock Marine Research, Gearhies, Bantry, Co. Cork, P75 AX07, Ireland. ^3^ International Iberian Nanotechnology Laboratory, Av. Mestre José Veiga, s/n, 4715-330, Braga, Portugal.

Email: [pilar.bermejo@usc.es](mailto:pilar.bermejo@usc.es)

**Contents**

Preparation of citrate NPs dispersions……………………………………. Page 2

Seaweed sample cultivation………………………………………………..Page 2

Table S1……………………………………………………………………Page 3

Table S2……………………………………………………………………Page 4

Table S3……………………………………………………………………Page 5

Table S4……………………………………………………………………Page 6

Fig.S1………………………………………………………………………Page 8

Fig.S2………………………………………………………………………Page 9

Fig.S3………………………………………………………………………Page 10

Fig.S4………………………………………………………………………Page 11

Fig.S5………………………………………………………………………Page 12

Fig.S6………………………………………………………………………Page 13

Fig.S7………………………………………………………………………Page 14

Fig.S8………………………………………………………………………Page 15

Fig.S9………………………………………………………………………Page 16

Fig.S10……………………………………………………………………...Page 17

**Preparation of citrate NPs dispersions**

Citrate-5 nm TiO_2_NPs and citrate-25 nm TiO_2_NPs dispersions were prepared by dispersing a mixture of trisodium citrate dihydrate (Sigma-Aldrich) and TiO_2_ powder at a weight ratio of TiO_2_: citrate 1:1.5 wt/wt for 5 nm and 1:0.8 wt/wt for 25 nm using an ultrasonic probe (Branson Disintegrator Ultrasonic Mod. 450) for 30 min (30 s pulse on /5 s pulse off, and 50 % amplitude). The final concentration of both citrate-TiO_2_NPs was 15.5 g L^-1^. The TiO_2_NPs stocks were diluted in 0.1 g mL^-1^ Cell-Hi F2P growing medium media (Varicon Aqua, Hallow, Worcester, UK) to reach 0.1 and 1.0 mg L^-1^ for bioaccumulation assays with seaweeds.

**Seaweed sample cultivation**

The seaweed's growth and feeding with the Cell-Hi F2P medium (a soluble nutrient blend commonly used in the production of marine microalgae) were taken care of for 28 days. Seaweeds were cultivated in 40 L tanks at 16±1 ºC with periods of 12 hours of white light and 12 hours of darkness. Parameters like pH, temperature, dissolved O_2_, and salinity were measured every day. However, conductivity, ammonium, and nitrites were tested twice a week. For each of the four trials, there were three control tanks (A-C: seaweed in growing medium without nanoparticles), three solvent control tanks (D-F: seaweed exposed to citrate, without nanoparticles), three low dose tanks (G-I: seaweed exposed to 0.1 mg L^-1^ of citrate-coated TiO_2_NPs), and three high dose tanks (J-L: seaweed exposed to 1.0 mg L^-1^ of citrate-coated TiO_2_NPs). 30 mL (0.1g mL^-1^) of the F2P growing medium containing the phytoplankton was added three times per week. Fifty percent of the water was replaced twice per week, two days before and two days after the addition of nanoparticles. The particles were added only once per week to the F2P medium by micro pipetting and manual shaking to obtain the final concentration (0.1 and 1.0 mg L^-1^) in the forty-liter tanks. Indigo Rock's staff collected three seaweed samples replicates from each tank on days 0, 7, 14, 21, and 28 for analysis by ICP-MS, TEM, and SEM. Three replicate samples from each tank and each trial for ICP-MS and SP-ICP-MS analysis of Ti and TiO_2_NPs were collected respectively, resulting in 180 samples per trial. The seaweed samples were washed with ultrapure water to remove salts and contaminants from the seawater. Wet seaweed samples were manually crushed, homogenized, and stored in polyethylene tubes at -18.0 ºC until microwave acid digestion and basic extraction of TiO_2_NPs were performed.

**Table S1.** Operational conditions for ICP-MS and SP-ICP-MS

| **Operational conditions ICP-MS** | | |
| --- | --- | --- |
| **Parameter/component** | **Type/mode** | **Value** |
| Nebulizer | Meinhard CR R^+^ | |
| Nebulizer Chamber | 5 ºC refrigerated glass cyclone chamber with Peltier PC^3X^ | |
| Cone material | Nickel/aluminum | |
| Radiofrequency power |  | 1600 W |
| Ar gas flow | Plasma | 15 L min^-1^ |
|  | Nebulizer | 1.15 L min^-1^ |
|  | Auxiliary | 1.2 L min^-1^ |
| Analyte | Ti | |
| Operation mode | DRC (dynamic reaction cell) | |
| Ammonia flow rate |  | 1.0 mL min^-1^ |
| Integration time |  | 1000 ms |
| m/z |  | 131, cluster [^48^Ti^14^N^1^H(^14^N^1^H_3_)_4_^+^] |
| Replicates per sample |  | 3 |
| RPQ |  | 0.2 |
| **Specific operational conditions for SP-ICP-MS** | | |
| Sample flow rate |  | 0.189-0.210 mL min^-1^ |
| Acquisition time |  | 60 s |
| Dwell time |  | 50 µs |
| Number of readings |  | 1.200.000 |

**Table S2.** Physicochemical characterization of selected TiO_2_NPs before exposure by DLS (n = 10) and zeta potential (n = 5), and after exposure and alkaline extraction by SP-ICP-MS (n=5)

| **Type of particle** | **Initial NPs dispersion in water** | | **Initial NPs dispersion in artificial seawater** | | **NPs Dispersion after exposure and extraction** | |
| --- | --- | --- | --- | --- | --- | --- |
|  | Hydrodynamic diameter/ nm | Zeta potential^2^/mV | Hydrodynamic diameter/ nm | Zeta potential/mV | Most frequent size/ nm | Mean size/ nm |
| Citrate-25 nmTiO_2_NPs | 128±2.9 | -79±2 | 3168±1089 | -1.4±2.4 | 37±1 | 52±1 |
| Citrate-5 nm TiO_2_NPs | 65±14 | -31±1 | 3320±1248 | -1.4±2.8 | 44±7 | 48±6 |

^1^ Mean hydrodynamic diameter obtained by DLS at a scattering angle of 173° and 25 °C. Ten DLS measurements were acquired: mean ± standard deviation (SD).

^2^ Zeta potentials were measured in 5 runs (mean ± SD).

**Table S3.** Total titanium content in *Palmaria palmata* and *Ulva* sp. exposed to TiO_2_NPs

|  | | ***Palmaria palmata*** | | | | ***Ulva* sp.** | | | |
| --- | --- | --- | --- | --- | --- | --- | --- | --- | --- |
|  |  | **Total content/ µg g^-1^** | | | | **Total content/ µg g^-1^** | | | |
| **NPs size/ nm** | | **25** | | **5** | | **25** | | **5** | |
| **Tanks/ Exposure time** | | **0 days** | **28 days** | **0 days** | **28 days** | **0 days** | **28 days** | **0 days** | **28 days** |
| **Control** | **A** | 0.30±0.08 | 0.43±0.01 | 0.28±0.03 | 0.27±0.05 | 0.28±0.08 | 0.15±0.04 | 1.38±0.62 | 0.28±0.16 |
|  | **B** | 0.17±0.07 | 0.45±0.01 | 0.26±0.05 | 0.35±0.02 | 0.43±0.08 | 0.40±0.17 | 1.08±0.27 | 0.19 |
|  | **C** | 0.24±0.05 | 0.51±0.01 | 0.36±0.01 | 0.34±0.01 | 0.16±0.07 | 0.18±0.02 | 0.25±0.05 | 0.40±0.10* |
| **Solvent** | **D** | 0.21±0.03 | 0.31±0.06 | 0.40±0.10 | 0.31±0.02 | 0.26±0.03 | 0.21±0.01 | 0.82±0.31 | 0.36±0.02 |
|  | **E** | 0.13±0.02 | 0.31±0.06 | 0.43±0.12 | 0.54±0.08 | 0.27±0.10 | 0.38±0.14 | 1.85±0.05 | 0.31±0.05 |
|  | **F** | 0.40±0.02 | 0.49±0.06 | 0.30±0.01 | 0.26±0.02 | 0.71±0.10 | 0.28±0.05 | 0.50±0.12 | 0.10±0.01 |
| **0.1 mg L^-1^** | **G** | 0.31±0.04 | 7.32±0.60 | 0.35±0.02 | 1.97±0.01 | 0.46±0.01 | 6.41±0.24 | 0.34±0.05 | 4.15±2.18 |
|  | **H** | 0.23±0.01 | 7.47±0.40 | 0.69±0.01 | 1.17±0.14 | 0.58±0.05 | 2.78±0.17 | 0.63±0.09 | 3.25±0.82 |
|  | **I** | 0.40±0.21 | 6.66±0.10 | 0.25±0.02 | 1.13±0.08 | 0.20±0.01 | 17.71±0.01 | 0.42±0.09 | 4.44±0.82 |
| **1.0 mg L^-1^** | **J** | 0.30±0.02 | 31.21±1.10 | 0.68±0.15 | 7.33±0.89 | 0.16±0.01 | 50.58±2.47 | 0.33±0.05 | 52.84±21.69 |
|  | **K** | 0.15±0.03 | 32.47±5.88 | 0.73±0.18 | 5.10±0.47 | 0.67±0.02 | 45.44±2.05 | 0.53±0.20 | 79.85±9.95 |
|  | **L** | 0.18±0.02 | 27.98±1.63 | 0.26±0.05 | 7.91±1.38 | 0.16±0.04 | 82.89±1.35 | 0.57±0.30 | 53.19±5.99 |

* 21 days of exposure

**Table S4.** TiO_2_NPs content, most frequent and mean size in *Palmaria palmata* and *Ulva* sp.

|  | | ***Palmaria palmata*** | | | | | | | | | | | |
| --- | --- | --- | --- | --- | --- | --- | --- | --- | --- | --- | --- | --- | --- |
|  | | **NPs content/ NPs g^-1^** | | | | **Most frequent size/ nm** | | **Mean size/ nm** | | **Most frequent size/ nm** | | **Mean size/ nm** | |
| **NPs size/ nm** | | **25** | | **5** | | **25** | | | | **5** | | | |
| **Tanks/ Exposure time/ days** | | **0** | **28** | **0** | **28** | **7** | **28** | **7** | **28** | **0** | **28** | **0** | **28** |
| **0.1 mg L^-1^** | **G** | - | 2.12×10^9^±9.27×10^7^ | - | - | 42±6 | 41±1 | 54±7 | 52±2 | - | - | - | - |
|  | **H** | 4.54×10^6^±4.01×10^5^ | 2.10×10^9^±1.58×10^8^ | - | - | 38±2 | 42±5 | 50±5 | 56±9 | - | - | - | - |
|  | **I** | - | 2.89×10^9^±1.71×10^8^ | - | - | 39±6 | 41±2 | 52±2 | 54±2 | - | - | - | - |
| **1.0 mg L^-1^** | **J** | - | 1.11×10^10^±5.17×10^8^ | - | 1.83×10^9^±1.02×10^8^ | 54±1 | 49±3 | 76±6 | 64±2 | 53±4 | 42±1 | 82±16 | 49±5 |
|  | **K** | - | 7.57×10^9^±1.36×10^9^ | 5.66×10^8^±2.38×10^8^ | 2.17×10^9^±4.16×10^8^ | 42±3 | 44±2 | 59±5 | 58±2 | 32±1 | 42±3 | 38±4 | 50±2 |
|  | **L** | - | 4.52×10^9^±5.71×10^8^ | 3.44×10^8^±1.07×10^8^ | 2.70×10^9^±4.36×10^8^ | 51±2 | 48±5 | 75±4 | 70±6 | 33±1 | 40±3 | 39±4 | 49±3 |
|  | | ***Ulva* sp.** | | | | | | | | | | | |
|  | | **NPs content/ NPs g^-1^** | | | | **Most frequent size/ nm** | | **Mean size/ nm** | | **Most frequent size/ nm** | | **Mean size/ nm** | |
| **NPs size/ nm** | | **25** | | **5** | | **25** | | | | **5** | | | |
| **Tanks/ Exposure time/ days** | | **0** | **28** | **0** | **28** | **0** | **28** | **0** | **28** | **0** | **28** | **0** | **28** |
| **0.1 mg L^-1^** | **G** | 2.74×10^8^±1.55×10^8^ | 1.64×10^9^±2.26×10^8^ | - | - | 34±2 | 47±3 | 51±5 | 58±6 | - | - | - | - |
|  | **H** | - | 1.90×10^9^±4.97×10^8^ | - | - | 38±9 | 36±1 | 50±6 | 48±1 | - | - | - | - |
|  | **I** | 4.53×10^8^±2.49×10^7^ | 4.25×10^9^±5.57×10^8^ | - | - | 36±3 | 45±4 | 54±2 | 55±4 | - | - | - | - |
| **1.0 mg L^-1^** | **J** | - | 8.59×10^9^±1.28×10^9^ | - | 5.59×10^9^±3.28×10^8^ | 42±2 | 36±6 | 53±13 | 49±9 | 51±8 | 55±2 | 71±18 | 65±3 |
|  | **K** | 5.31×10^8^±9.47×10^7^ | 5.56×10^9^±5.19×10^8^ | - | 5.01×10^9^±8.32×10^7^ | 38±2 | 62±5 | 44±3 | 77±8 | 46±4 | 51±1 | 80±11 | 64±2 |
|  | **L** | 6.47×10^8^±1.78×10^7^ | 5.07×10^9^±2.53×10^8^ | - | 5.62×10^9^±1.53×10^9^ | 51±1 | 57±8 | 61±1 | 72±6 | 41±4 | 49±5 | 77±4 | 63±6 |

- Indicate <LOD


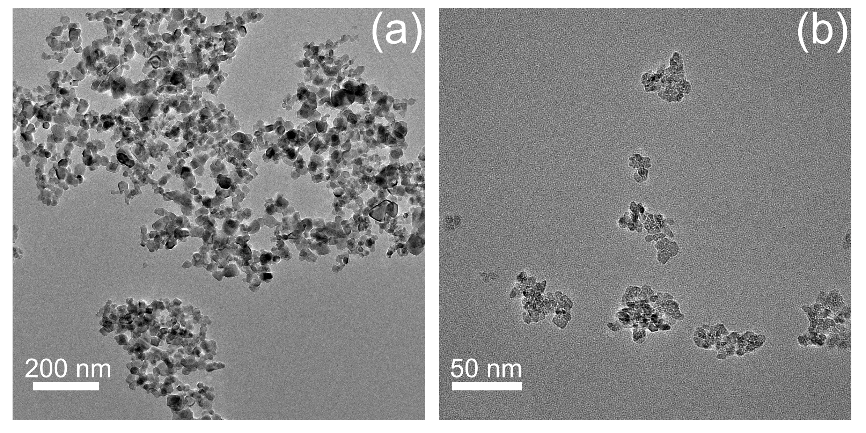


**Fig. S1.** Representative TEM images of (a) citrate-25 nm TiO_2_NPs and (b) citrate-5 nm TiO_2_NPs.





**Fig. S2.** X-ray diffraction pattern of anatase 5 nm TiO_2_NPs. The nano crystalline size was calculated using Scherrer equation *L=Kλ/βcosθ* (*L =* nano crystallite size; *λ (nm)* = XRD radiation of wavelength; *β* = full width at half maximum of peaks; *θ* = peak centered at 2*θ* in the pattern). The crystalline nanosize obtained was 8.6 nm.


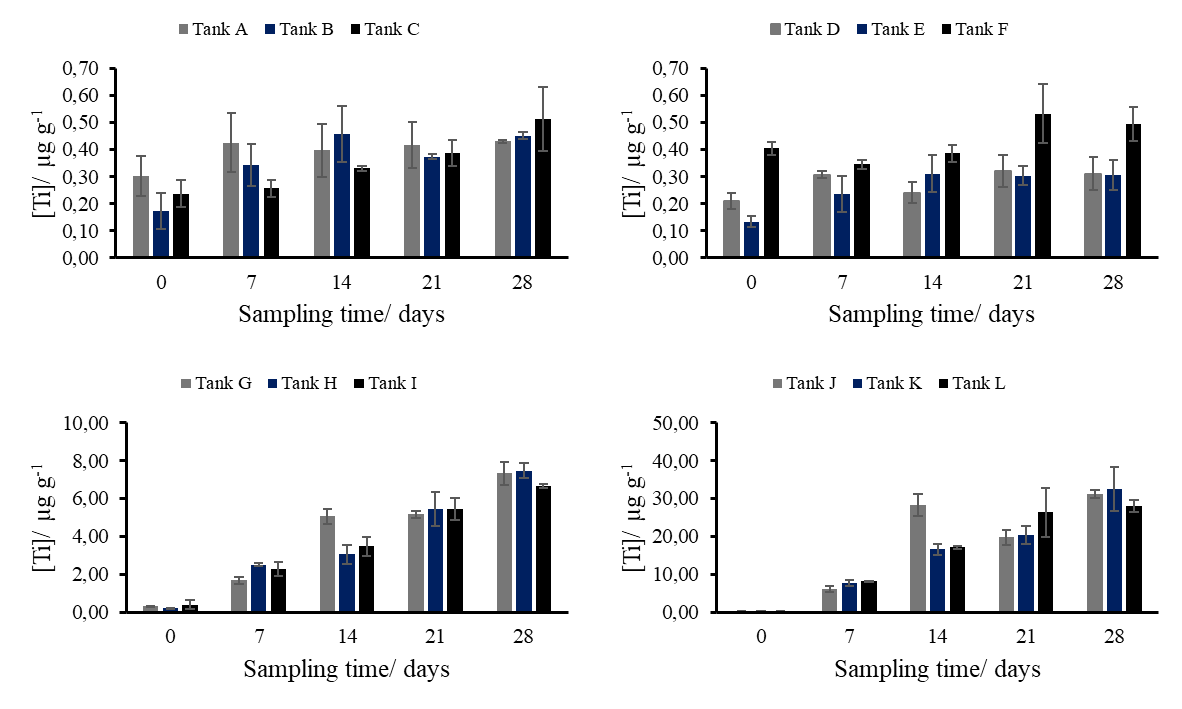


**Fig. S3.** Total content of Ti in *Palmaria palmata* grown in: (a) control tanks, (b) solvent tanks, (c) tanks with 0.1 mg L^-1^ of 25 nm TiO_2_NPs, and (d) tanks with 1.0 mg L^-1^ of 25 nm TiO_2_NPs**.**


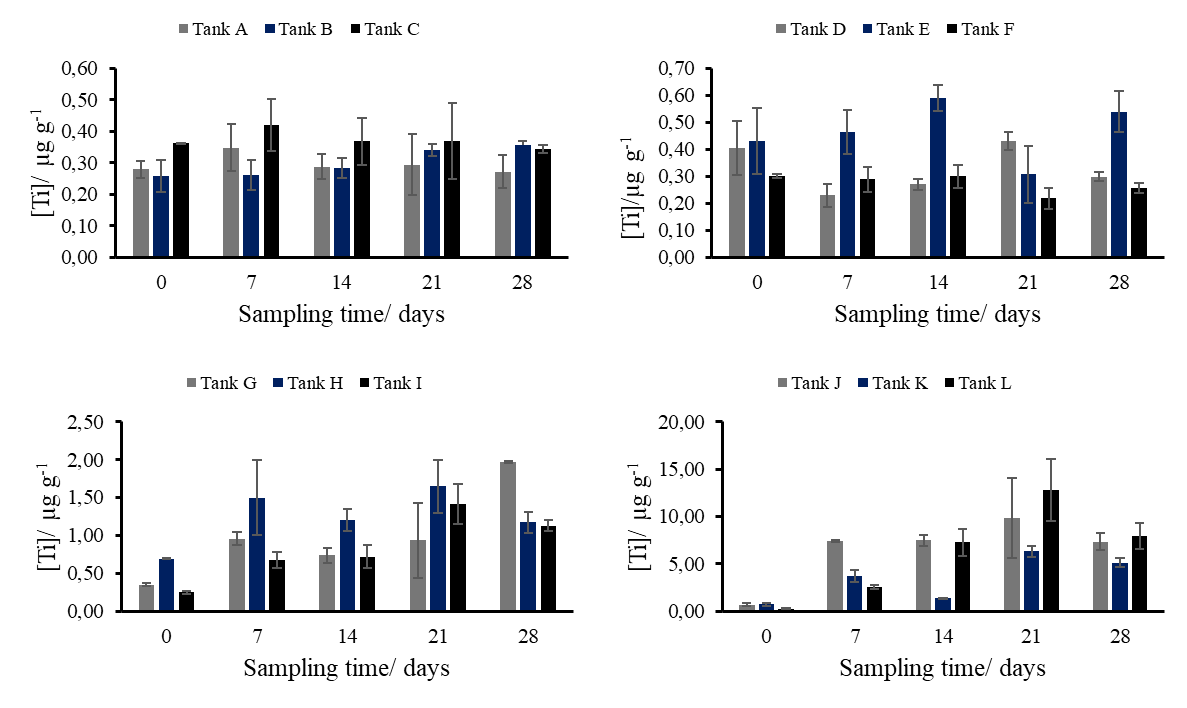


**Fig. S4.** Total content of Ti in *Palmaria palmata* grown in: (a) control tanks, (b) solvent tanks, (c) tanks with 0.1 mg L^-1^ of 5 nm TiO_2_NPs, and (d) tanks with 1.0 mg L^-1^ of 5 nm TiO_2_NPs.


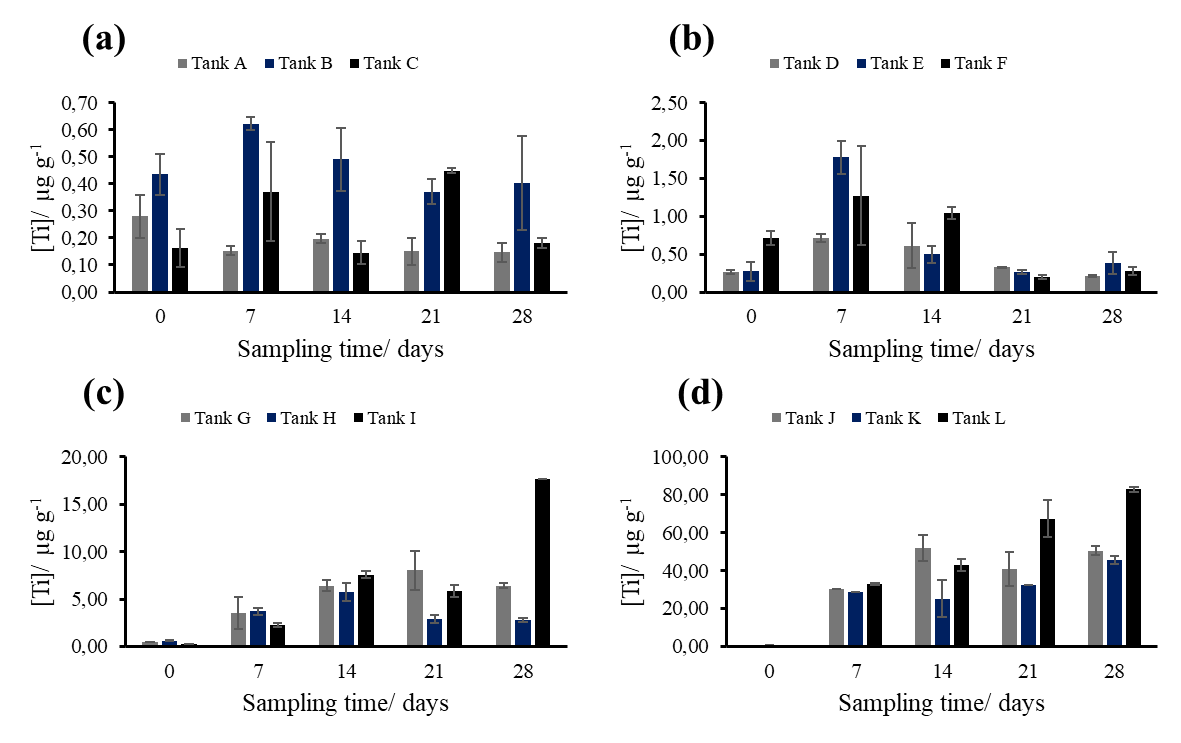


**Fig. S5.** Total content of Ti in *Ulva* sp. grown in: (a) control tanks, (b) solvent tanks, (c) tanks with 0.1 mg L^-1^ of 25 nm TiO_2_NPs, and (d) tanks with 1.0 mg L^-1^ of 25 nm TiO_2_NPs.


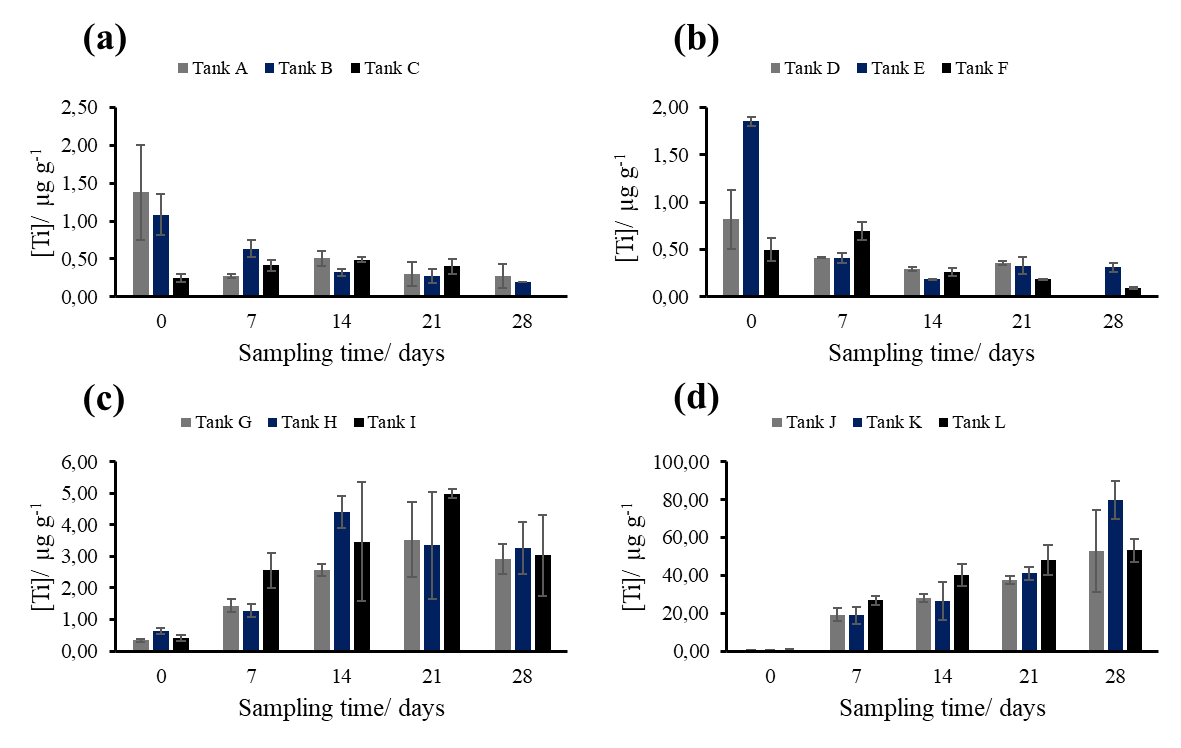


**Fig. S6.** Total content of Ti in *Ulva* sp. grown in: (a) control tanks, (b) solvent tanks, (c) tanks with 0.1 mg L^-1^ of 5 nm TiO_2_NPs, and (d) tanks with 1.0 mg L^-1^ of 5 nm TiO_2_NPs.

**Fig. S7.** Content of TiO_2_NPs g^-1^ in *Palmaria palmata* exposed to: (a) 0.1 mg L^-1^ of 25 nm TiO_2_NPs, (b) 1.0 mg L^-1^ of 25 nm TiO_2_NP, and (c) 1.0 mg L^-1^ of 5 nm TiO_2_NPs.


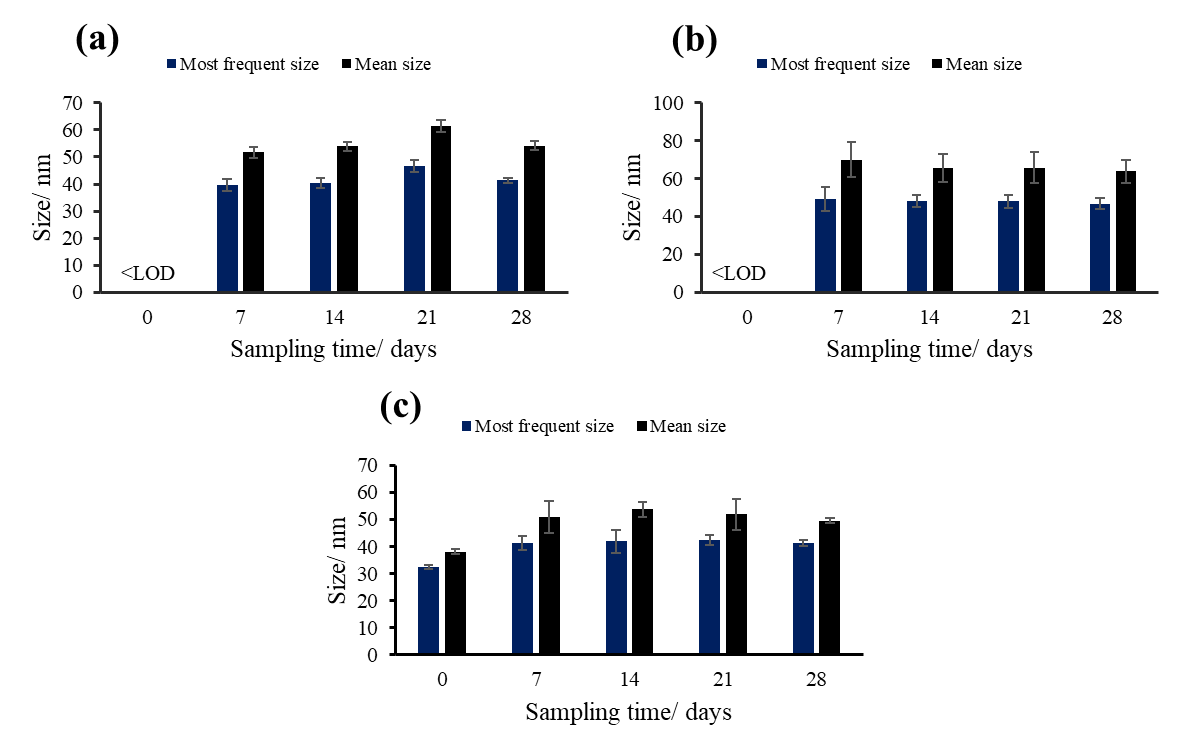


**Fig. S8.** Most frequent and mean sizes of nanoparticles in *Palmaria palmata* exposed to: (a) 0.1 mg L^-1^, (b) 1.0 mg L^-1^ of 25 nm TiO_2_NPs, and (c) 1.0 mg L^-1^ of 5 nm TiO_2_NPs.


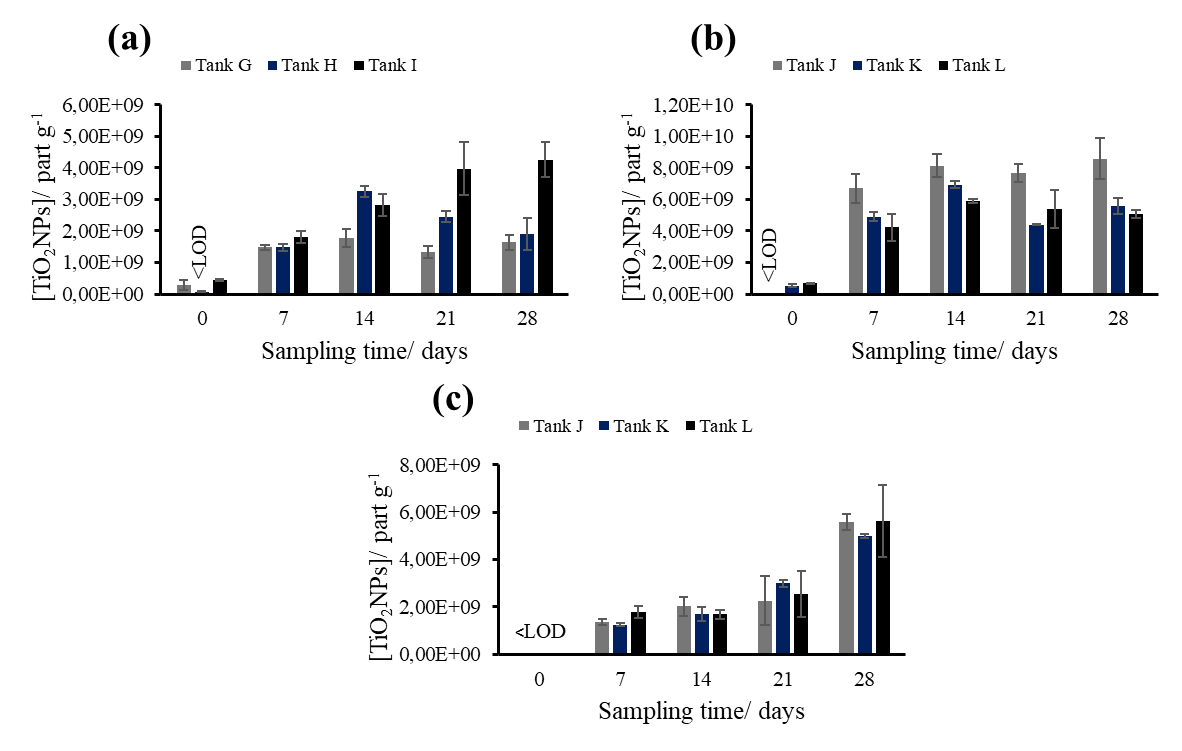


**Fig. S9.** Content of TiO_2_NPs g^-1^  in *Ulva* sp. exposed to: (a) 0.1 mg L^-1^, (b) 1.0 mg L^-1^ of 25 nm TiO_2_NPs, and (c) 1.0 mg L^-1^ of 5 nm TiO_2_NPs.


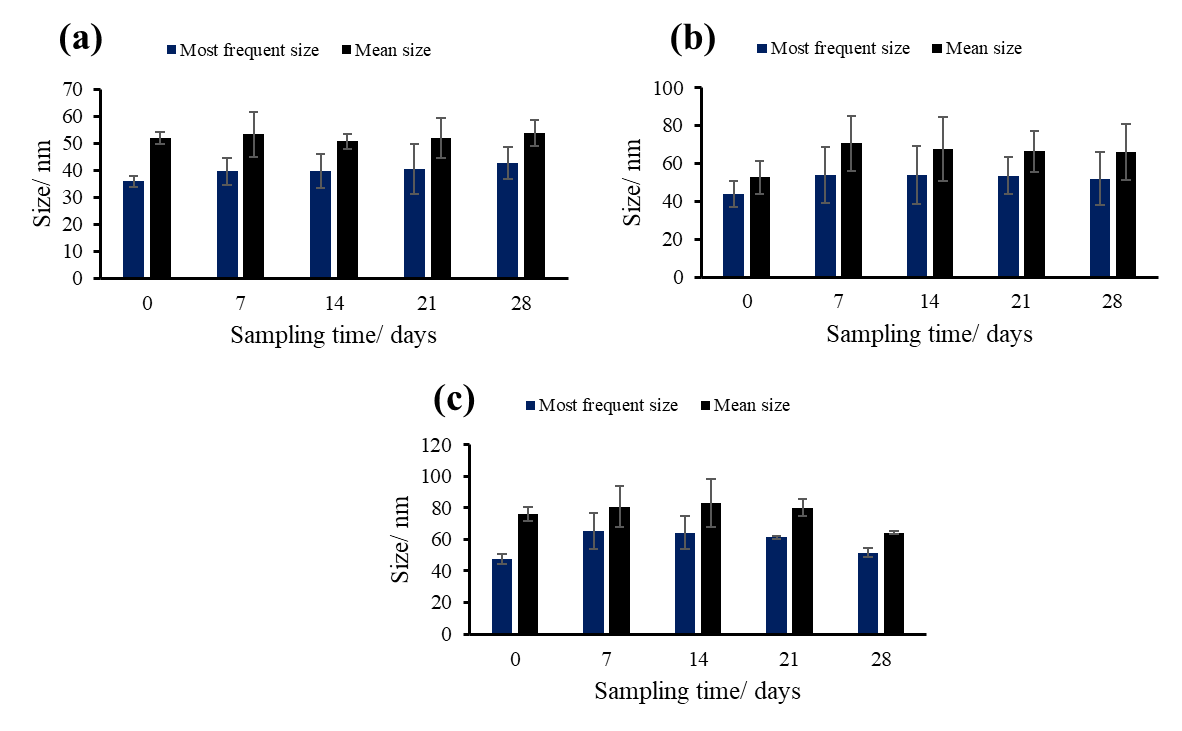


**Fig. S10.** Most frequent and mean sizes of nanoparticles in *Ulva* sp. exposed to: (a) 0.1 mg L^-1^, (b) 1.0 mg L^-1^ of 25 nm TiO_2_NPs, and (c) 1.0 mg L^-1^ of 5 nm TiO_2_NPs.
